# Recidivism rates of female offenders discharged from forensic psychiatric treatment

**DOI:** 10.3389/fpsyt.2025.1556987

**Published:** 2025-03-27

**Authors:** Juliane Mayer, Viviane Wolf, Ivonne Steiner, Manuela Dudeck, Verena Klein, Judith Streb, Irina Franke

**Affiliations:** ^1^ Department of Forensic Psychiatry and Psychotherapy, kbo Kliniken des Bezirks Oberbayern, Munich, Germany; ^2^ Department of Forensic Psychiatry and Psychotherapy, Ulm University, Ulm, Germany; ^3^ Department of Psychiatry and Psychotherapy, LVR Klinik Düsseldorf, Düsseldorf, Germany; ^4^ Psychiatric Services of Grisons, Chur, Switzerland

**Keywords:** recidivism rates, female offenders justice-involved women, forensic psychiatric treatment, mental disorders, substance use disorders, risk assessment

## Abstract

**Objective:**

Recidivism rates comprise an essential component in comprehensive risk assessment and should reflect the specific reference group of the individual being assessed. For female offenders with mental disorders, recidivism rates are nearly nonexistent. The goal of this study is to report offense- and disorder-related recidivism rates for the understudied group of female offenders discharged from forensic psychiatric treatment.

**Method:**

The sample consisted of 525 German patients released from placement orders according to Section 63 (*n* = 110) or 64 of the German Criminal Code (*n* = 415), indicating a diagnosis of a serious mental disorder or substance use disorder, respectively. In a retrospective design, we analyzed archived patient files as well as official reconviction records.

**Results:**

With average times at risk of 8.5 and 5.3 years for each placement order, we observed general recidivism rates of 19% and 46%, and violent recidivism rates of 8% and 12%. Offense-related recidivism rates showed high numbers for property offenders, threateners, and arsonists. Disorder-related recidivism rates revealed that a comorbidity of schizophrenia and alcohol use disorder increased the risk of general reoffending eightfold (Exp[*B*] = 8.167; *p* = .025), while a comorbid substance use disorder and personality disorder heightened the violent recidivism risk fourfold (Exp[*B*] = 4.204; *p* = .029). Subgroup analysis of patients with substance use disorders indicated that treatment dropouts were about three times more likely to recidivate than patients who completed treatment (Exp[*B*] = 2.863; *p* <.001).

**Conclusion:**

The results provide rare recidivism data for risk assessment of female offenders with mental disorders and underscore the protective effect of forensic psychiatric treatment, including forensic aftercare, on recidivism.

## Introduction

Risk assessment is concerned with assessing an offender’s risk of reoffending and informs a wide range of decisions as well as treatment and risk management strategies within the criminal justice system (1). When it comes to incarcerated offenders, risk assessment particularly serves as a basis for decision-making on the granting of furloughs or conditional releases (2).

The fundamental group statistic in risk assessment is the recidivism rate, which is the statistical prevalence of reoffending in a given reference group of offenders in a given time (3). Recidivism rates are incorporated in multiple risk assessment instruments, which employ evidence-based risk factors for recidivism to individualize an offender’s risk of reoffending (4). A German interdisciplinary task force specifically recommends the inclusion of recidivism rates of specific reference groups, most commonly offense-related recidivism groups, as a crucial part of an empirically informed and scientifically based risk assessment (5).

When it comes to offenders with mental disorders discharged from forensic psychiatric treatment, international recidivism rates vary greatly. A rare meta-analysis showed that general reoffending rates range from 0 to 24,244 per 100,000 person-years, while violent reoffending rates range from 273 to 8,403 per 100,000 person-years (6). These variations can partly be attributed to differences in forensic psychiatric populations, their diagnoses, and comorbidities (e. g., substance use disorders and personality disorders), as well as treatment interventions (7). Overall, the authors reported lower recidivism rates for both general and violent reoffending compared to other offender groups, concluding that forensic psychiatric treatment is effective in reducing recidivism (26).

In Germany, placement in a forensic psychiatric hospital is based on a court decision according to Section 63 or 64 of the German Criminal Code. The aim of the placement in both cases is preventing recidivism by securing offenders with mental disorders as well as providing adequate treatment (9). If a person commits a serious criminal offense due to a severe mental disorder and there is a high risk of recidivism, the court orders the person to be hospitalized according to Section 63 of the German Criminal Code (Section 63). There is no legal limit to the length of hospitalization. However, annual risk assessments and hearings on discharge readiness are required by the court. Here, a clinical report of a positive change in the patient’s mental status and a reduction in the risk of recidivism prompts conditional release.

Placement under Section 64 of the German Criminal Code (Section 64) requires a substance use disorder being attributed to the index offense, a high risk of reoffending, and a favorable treatment prognosis. With a standard treatment duration of two years, biannual risk assessments and hearings on discharge readiness are required by the court. While a clinical report of a positive change in the patient’s mental status and a reduction in the risk of recidivism allow for a conditional release, treatment can be terminated if a favorable treatment prognosis is no longer tenable. In the latter case, patients usually serve the remainder of their sentence in prison.

Patients placed according to Sections 63 or 64 are treated on separate wards in specialized secure forensic psychiatric hospitals. Upon regular discharge, patients are placed on conditional release and must comply with mandatory treatment und supervision orders, such as outpatient care and treatment, substance monitoring, supportive living, and regular employment, which are usually enforced by specialized forensic aftercare services.

For both placement orders, risk assessment plays a crucial role for the justification of the continuation or termination of the treatment. Here, specified recidivism rates gain significance as these offender groups differ from the general population of prisoners due to having a psychiatric diagnosis - including psychotic, personality, and substance use disorders - that is oftentimes related to a higher risk of general and violent offending, particularly if untreated [e. g., (10)]. In the past, nationwide recidivism studies on forensic patients after discharge have been rare and mostly provided data on the total group of discharged patients. Jehle et al. (11) reported that 13% and 48% of patients released from placement according to Sections 63 (*n* = 836) and 64 (*n* = 2,164) (gender distributions not reported), respectively, recidivated within a three-year period post discharge. However, they did not provide nationwide recidivism data for patients accounting for different offense groups and diagnoses, when it would be highly relevant for risk assessment due to the heterogeneity of patients (12). Also, no data on follow-up periods beyond three years were reported.

In recent years, several studies in different German federal states or governmental districts tried to overcome this gap in empirical knowledge. In the context of the Munich project on risk assessment, Stadtland and Nedopil (13) reported disorder-related recidivism rates in a sample of offenders participating in forensic psychiatric evaluation (*N* = 118, 84.9% male). For patients released from placement according to Section 63, the Essen long-term study (12, 14) provided the only German prospective long-term data so far, following up on 321 patients (94% male) released from 23 forensic hospitals in seven federal states for an average period of 16.5 years. They found recidivism rates of 35% and 13% for general and violent reoffending, respectively, and reported more detailed data on recidivism according to index offenses (homicide, assault, sex offenses, property crimes, arson, other) and diagnoses (schizophrenia, personality disorders, organic disorders, intellectual disability). Patients with homicide as their index offense had the lowest recidivism rate with 22.5%, while sex offenders who did not use violence had the highest recidivism rate with 58.1% (12). When it comes to diagnoses, patients with organic disorders recidivated at the lowest rate (17.7%), while patients with personality disorders showed the highest recidivism rate (58.9%; 13). Overall, recidivism rates were lower compared to prison populations and reoffenses committed were less severe than index offenses (12).

For patients released from placement according to Section 64, there are even less specified data on recidivism rates available. Gericke and Kallert (15) reported a recidivism rate of 36.7% within two years after regular discharge for 120 patients (gender distribution not reported) placed in Saxony, with property crime offenders recidivating at the highest rate (27.3%) among all recidivists. A study with 449 patients (gender distribution not reported) from Mecklenburg-Vorpommern with a longer average follow-up-period of 5.8 years showed that 58.6% recidivated (16). They also detailed recidivism rates based on index offenses: 71% for violent offenders, 67% for property crime offenders, and 43% for other (non-violent) offenders. Additionally, they found higher recidivism rates and more severe reoffending among patients who dropped out of treatment prematurely. Similarly, Querengässer et al. (17) showed for 261 patients (94% male) in Baden-Wuerttemberg over an average follow-up period of 50 months that treatment dropouts recidivated faster and more severely as well as at a higher rate (72.8%) than patients who completed treatment regularly (48.6%). Although only about 50% of patients placed under Section 64 complete treatment regularly, recidivism rates for patients terminating treatment and returning to prison are usually not reported by design (e. g., 8). However, an elaborate German study performing a direct comparison with a carefully matched sample of prisoners found that the estimated rate of new convictions after 1000 days at risk was 47% in the treatment group (n = 314) and 67% in the control group of prisoners (n = 314), indicating an absolute risk reduction of 20% for male forensic patients (18). Obviously, recidivism rates differ vastly across federal states, which is partly accounted for by the fact that the legal foundations of treatment during a placement order are laid down in state laws (19).

In the studies cited above, women represent only a small percentage of patients or are not reported on at all. In Germany, women comprise only 5.7% of all inmates (20) and have a lower general recidivism rate (26%) than men (37%; 10), which is also reported in international studies [e. g., (7, 10)]. While there has been a growing research focus on female offender populations in the last two decades, fueled by an increase in the number of women entering criminal justice systems (21), the phenomenon of the “gender gap” in offending has been consistently observed across time, cultures, and countries (22). Consequently, this disparity has led to a proliferation of research on risk assessment and recidivism rates that almost exclusively concentrates on men (23).

However, the prevalence of justice-involved women has been on the rise in the past two decades (24). In Germany, the proportion of women treated in forensic psychiatric hospitals grew from 4.6% in 1975 to 7.4% in 2014 (25). Still, given the relatively small number of women in forensic psychiatric treatment, studies on female forensic patients and reoffending are scarce and face methodical challenges, such as small sample sizes, self-reports and short follow-up periods (26). De Vogel et al. (8) found general and violent recidivism rates of 33.8% and 18.3%, respectively, for 78 women discharged from forensic psychiatric treatment in the Netherlands. While Köhler (27) provided detailed recidivism data on female prisoner populations, to our knowledge, only two studies reported recidivism rates of female offenders released from forensic psychiatric treatment in Germany: Frey analyzed data on treatment outcome in 27 female patients placed according to Section 64 in four forensic treatment facilities and found that 37% of the sample recidivated. Franke et al. (9) compared male and female patient characteristics and the outcome of forensic treatment according to both Sections 63 and 64 in three forensic psychiatric hospitals in Bavaria. No female patient placed according to Section 63 (*n* = 50) recidivated one year after discharge compared to 9% of women placed according to Section 64 (*n* = 72), with 32 women being excluded from analyses beforehand due to premature treatment dropout. While they also showed that 50% of reoffenses committed by women were in the same category as the index offense and none more serious than the index offense, the study design (short follow-up period, medium sample sizes) did not allow for further specification of recidivism rates according to index offenses or diagnoses.

The present study was conducted at the Department of Forensic Psychiatry and Psychotherapy of the kbo-Isar-Amper-Hospital Taufkirchen (Vils), Bavaria, the only forensic psychiatric hospital in Germany exclusively for women. This enabled recourse to a much larger sample size as well as a longer follow-up period for recidivism research on the otherwise underrepresented subgroup of female offenders with mental disorders. The hospital offers a variety of specialized treatment programs, including pharmacological treatment, psychotherapy (individual and group therapy, e. g., dialectic behavior therapy), nonverbal forms of therapy (e.g., art therapy), work therapy, and school education (28). Subsequent forensic aftercare offers support with managing medication, living and work conditions, as well as relapses and psychiatric crises through regular appointments or home visits.

The aim of the present study is to report detailed recidivism rates according to index offenses and diagnoses for female offenders released from forensic psychiatric treatment for both Sections 63 and 64. Moreover, for patients placed according to Section 64, recidivism rates between patients who completed their treatment regularly and patients who dropped out prematurely are compared. This allows for a rare opportunity to analyze the efficacy of the treatment with treatment dropouts serving as an approximate control group (17). The results might not only contribute to knowledge on the efficacy of forensic psychiatric treatment in terms of recidivism rates but also provide empirical data for appropriate risk assessment of the otherwise understudied group of female offenders with mental disorders.

## Materials and methods

### Procedure

In a retrospective design, patient data were collected from archived patient records, including official court documents. Based on an extensive literature review on gender-specific risk factors for recidivism, we designed a codebook in collaboration with the Office of Corrections and Rehabilitation, Zurich, Switzerland. The study assessed sociodemographic variables (e. g., age), criminological variables (e. g., index offense), clinical variables (e. g., age of onset), and treatment variables (e. g., problematic incidents). Diagnoses were coded at discharge according to the diagnostic criteria of the ICD-10. Offense severity was categorized according to the Cormier-Lang system (29), which captures the frequency and severity of offenses ranging from 1 (minor property offense) to 28 (homicide). Patient consent was waived because data was analyzed retrospectively in a way that did not allow identification. An ethics vote for this study was obtained from the Medical Association (protocol no. 2019-167).

### Patient sample

The sample included all patients who were legally admitted to the forensic psychiatric hospital in Taufkirchen (Germany) and discharged between January 1, 2001, and December 31, 2017. In total, data records of 557 women were collected. After excluding 32 incomplete records from further analysis, the final sample comprised 525 patients, with 110 patients placed according to Section 63 and 415 patients placed according to Section 64. Time at risk, i. e., the follow-up period for recidivism from the date of hospital discharge (for treatment dropouts from the date of prison release) until the first reoffense or the end date of the survey (if no reoffense occurred), was on average 6 years (*SD* = 4.9) and ranged from 2 months to 19 years. **Tables 1**, **2** present descriptive data for patients placed according to Sections 63 and 64, respectively.

**Table 1:** Descriptive statistics for patients placed according to Section 63.

	*n*	Freq. (*n*)	%	Mean	*SD*
Age at discharge (years)	110	-	-	46.01	12.055
Relationship status at discharge	110				
Single	81	73.6			
Married	8	7.3			
Stable relationship/de-facto marriage	12	10.9			
Unstable relationship	9	8.2			
Main diagnosis at discharge	109				
Organic mental disorders (F0)		5	4.5		
Schizophrenia spectrum disorders (F2)		79	71.8		
Mood or neurotic, stress-related disorders (F3-F4)		6	5.5		
Emotionally unstable personality disorder (F60.3)		12	10.9		
Mixed and other personality disorders (F60.2, F61)		3	2.7		
Intellectual disability (F70)		1	.9		
Conduct disorders (F91)		3	2.7		
Age of onset of psychiatric symptoms (years)	110	-	-	20.41	12.379
Violence during treatment	110	37	33.6	-	-
Other antisocial behavior during treatment	110	69	62.7	-	-
Duration of treatment (years)	110	-	-	6.01	3.237
Index offense	110				
Public order offense		1	.9		
Traffic offense, drunken stupor		2	1.8		
Property offense		14	12.7		
Resistance to state authority		1	.9		
Coercion, abduction, threat		6	5.5		
Robbery, extortion		5	4.5		
Arson		19	17.3		
Assault		37	33.6		
Homicide, manslaughter		25	22.7		
Violent index offense	110	93	84.5	-	-
Index offense severity	110	-	-	9.47	9.259
Sentence (months)	25	-	-	28.16	19.459
Age at first conviction (years)	110	-	-	36.32	13.249
Number of previous convictions	110	-	-	1.93	3.202
Time at risk (years)	110	-	-	8.50	4.511

*SD*, standard deviation; Freq., frequency.

**Table 2:** Descriptive statistics for patients placed according to Section 64.

	*n*	Freq. (*n*)	%	Mean	*SD*
Age at discharge (years)	415	-	-	35.52	9.207
Relationship status at discharge	413				
Single		225	54.2		
Married		26	6.3		
Stable relationship/de-facto marriage		57	13.7		
Unstable relationship		105	25.3		
Main diagnosis at discharge	415				
Disorders due to use of alcohol (F10)		71	17.1		
Disorders due to use of opioids (F11)		73	17.6		
Disorders due to use of other substances (F12-14)		17	4.1		
Disorders due to use of other stimulants (F15)		38	9.2		
Disorders due to multiple drug use (F19)		194	46.7		
Schizophrenia spectrum disorders (F2)		3	.7		
Mood disorders (F3)		1	.2		
Emotionally unstable personality disorder (F60.3)		13	3.1		
Mixed personality disorders (F61)		5	1.2		
Age of onset of psychiatric symptoms (years)	415	-	-	14.31	6.725
Drug substitution	364	138	50.3		
Relapses during treatment	415	133	32.0	-	-
Violence during treatment	415	40	9.6	-	-
Other antisocial behavior during treatment	415	228	54.9	-	-
Duration of treatment (years)	415	-	-	2.27	1.121
Index offense	415				
Sexual coercion		1	.2		
Defamation, false allegation		1	.2		
Traffic offense, drunken stupor		17	4.1		
Property offense		61	14.7		
Resistance to state authority		1	.2		
Coercion, abduction, threat		3	.7		
Robbery, extortion		24	5.8		
Narcotics offense		201	48.4		
Arson		10	2.4		
Assault		75	18.1		
Homicide, manslaughter		21	5.1		
Violent index offense	415	144	34.7	-	-
Index offense severity	415	-	-	3.45	5.338
Sentence (months)	401	-	-	34.53	21.129
Age at first conviction (years)	414	-	-	25.45	9.201
Number of previous convictions	415	-	-	4.81	4.287
Time at risk (years)	415	-	-	5.30	4.472

*SD*, standard deviation; Freq., frequency.

### Recidivism

Information on criminal recidivism was obtained from the German Federal Office of Justice, which provided extracts on convictions entries in the Federal Central Criminal Register, in September 2020 and, due to missing data, again in February 2021 (end dates of survey). Three binary measures (i. e., yes/no) were used for general recidivism, defined as a conviction for any new offense, violent recidivism, defined as a conviction for an offense involving crimes against persons (e.g., homicide, sex crimes, arson, assault, threat, and robbery), and relevant recidivism, defined as a conviction for an offense in the same category as the index offense. Only convictions committed and sentenced after release were assessed as a reoffense.

### Statistics

Statistical analyses were performed using IBM SPSS Statistics Version 29. For descriptive statistics and recidivism rates, absolute and relative frequencies, mean values, standard deviations, and ranges were calculated. Differences between treatment dropouts and regularly discharged patients were analyzed by Pearson’s chi-square (*χ²*) tests and Mann Whitney *U* tests, as means were not normally distributed. Cramer’s *V* was used as a measure of effect size, with effect sizes below .10 being interpreted as small, those between .10 and .30 as medium, and those above .30 as large (30). Wilcoxon ranks test was performed to compare offense severity. Kaplan-Meier survival curves were plotted to assess recidivism rates over time. The survival curves included all patients, i. e., patients who did not recidivate within the time at risk were included as censored cases. Cox regression analyses were performed to compare the risk of recidivism in the analyzed groups over the time at risk. Binary logistic regression analyses were conducted to determine the specific contribution of comorbidities in predicting recidivism. A *p*-value below .05 was deemed indicative of statistical significance.

## Results

### Overall recidivism rates

About 19.1% of patients (*n* = 21) placed according to Section 63 recidivated within a mean time at risk of 8.5 years. Less than half of the recidivists committed violent reoffenses (*n* = 9), resulting in a violent recidivism rate of 8%. The recidivism rate for reoffenses relevant to the index offense was 11% in this sample (*n* = 12).

In the sample of patients placed according to Section 64, 45.1% (*n* = 187) committed new offenses within a mean time at risk of 5.3 years. The violent recidivism rate was 12.3% with 51 patients committing new violent offenses. Relevant reoffenses were committed by 25.3% of patients in this group (*n* = 105).

**Figure 1:**
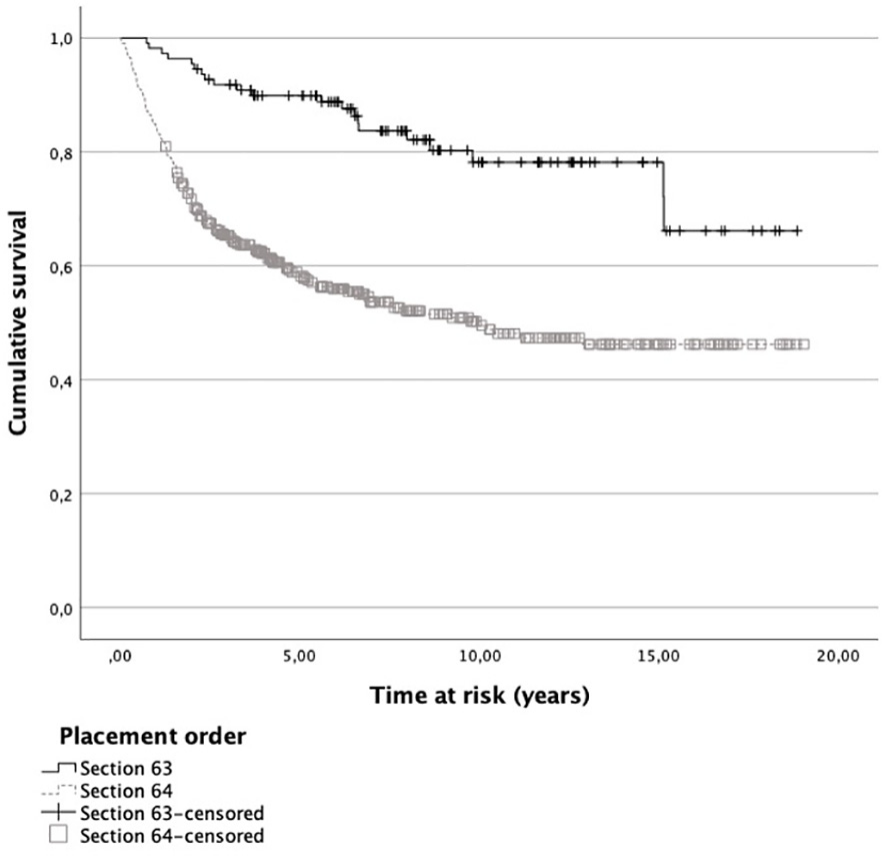
Survival functions for general recidivism of patients placed according Sections 63 and 64. Note. Patients who did not recidivate within the time at risk are included as censored cases.

Throughout the time at risk, survival functions for general recidivism showed that 98.2% and 84.6% of patients placed according to Sections 63 and 64, respectively, did not recidivate within one year after discharge (see **Figure 1**). After five years, the survival rate dropped to 89.9% and 58.5%, respectively. Cox regression analysis showed that patients placed according to Section 64 were about 3.3 times more likely to reoffend in the time at risk than patients placed according to Section 63 (Exp[*B*] = 3.271; *p* <.001).

Regarding the sentencing for the first reoffense, most patients in both samples were sentenced to a money fine (see **Table 3**). One third of recidivists in the group of patients placed according to Section 63 received a new placement order. In the sample of patients placed according to Section 64, about 21% of recidivists got a prison sentence and another 20% a suspended prison sentence for the first reoffense. About 5% were given a new placement order.

**Table 3:** Type of sentencing for the first reoffense.

Type of sentencing	Section 63 (*n* = 110)		Section 64 (*n* = 415)	
	Frequency	%	Frequency	%
No indictment due to diminished responsibility	-	-	2	.5
Money fine	12	10.9	97	23.4
Suspended sentence	2	1.8	37	8.9
Prison sentence	-	-	40	9.6
Placement according to Section 63	7	6.3	2	.5
Placement according to Section 64	-	-	8	1.9
Other	-	-	1	.25
Total	21	19.1	187	45.1

### Offense-related recidivism rates

Within the sample placed according to Section 63, patients who committed property offenses as well threats generated the highest general recidivism rates with 53.3% and 50%, respectively (see **Table 4**). Considerably lower rates were found for patients who committed violent offenses like assault (21.6%), robbery (20%), and arson (10.5%) as their index offense. Patients convicted of homicide did not recidivate, although they comprised 22.7% of the sample (see **Table 1**).

**Table 4:** General recidivism rates with respect to the index offense.

Index offense	Section 63			Section 64		
	*n*	Frequency	%	*n*	Frequency	%
Sexual coercion	0	-	-	1	0	0
Defamation, false allegation	0	-	-	1	1	100
Public order offense	1	0	0	0	-	-
Traffic offense	2	0	0	7	5	71.4
Drunken stupor	0	-	-	10	5	50
Property offense	15	8	53.3	61	34	55.7
Resistance to state authority	1	0	0	1	1	100
Coercion, kidnapping	3	1	33.3	3	2	66.7
Threat	2	1	50	0	-	-
Robbery, extortion	5	1	20	24	13	54.2
Narcotics offense	0	-	-	201	74	36.8
Arson	19	2	10.5	10	6	60
Assault	37	8	21.6	75	39	52
Homicide, manslaughter	25	0	0	21	7	33.3
Total	110	21	19.1	415	187	45.1

Within the sample placed according to Section 64, patients who committed narcotics offenses and homicide had the lowest recidivism rates with 36.8% and 33.3%, respectively (see **Table 4**). The single patients convicted of false allegation and resistance to state authority both recidivated, yielding a 100% general recidivism rate for these index offenses. Patients convicted of a traffic offense recidivated with 71.4%. More than half of the patients with other index offenses recidivated, while the patient convicted of sexual coercion did not.

Regarding violent recidivism in patients placed according to Section 63, 50% of patients who committed threats and 33.3% of patients who were convicted of coercion or kidnapping reoffended with another violent crime (see **Table 5**). However, we found no statistically significant correlation between a violent index offense and reoffense in this group (Cramer’s *V* = .036, *p* = .707).

**Table 5:** Violent recidivism rates with respect to the index offense.

Index offense	Section 63			Section 64		
	*n*	Frequency	%	*n*	Frequency	%
Sexual coercion	0	-	-	1	0	0
Defamation, false allegation	0	-	-	1	0	0
Public order offense	1	0	0	0	-	-
Traffic offense	2	0	0	7	1	14.3
Drunken stupor	0	-	-	10	2	20
Property offense	15	1	6.7	61	6	9.8
Resistance to state authority	1	0	0	1	0	0
Coercion, kidnapping	3	1	33.3	3	0	0
Threat	2	1	50	0	-	-
Robbery, extortion	5	1	20	24	3	12.5
Narcotics offense	0	-	-	201	11	5.5
Arson	19	1	5.3	10	6	60
Assault	37	4	10.8	75	18	24
Homicide, manslaughter	25	0	0	21	4	19.0
Total	110	9	8.1	415	51	12.3

Arsonists placed according to Section 64 recidivated at the highest rate (60%) with a violent reoffense. Less surprisingly, 24% of patients who committed assault and 19% of patients convicted of a capital offense recidivated with a violent crime (see **Table 5**). The correlation between a violent index offense and a violent reoffense showed to be statistically significant with a medium-sized effect (Cramer’s *V* = .236, *p* <.001) in patients placed according to Section 64.

In both patient samples, property offenders had among the highest recidivism rates for relevant reoffenses (see **Table 6**). Somewhat surprisingly, only 21.9% of patients convicted for a narcotics offense recidivated with a new drug offense, whereas 57.1% of patients convicted for traffic offenses committed a relevant reoffense within the sample of patients placed according to Section 64. Two patients convicted of homicide recidivated with a relevant offense in that the index offense included assault charges.

**Table 6:** Relevant recidivism rates with respect to the index offense.

Index offense	Section 63			Section 64		
	*n*	Frequency	%	*n*	Frequency	%
Sexual coercion	0	-	-	1	0	0
Defamation, false allegation	0	-	-	1	0	0
Public order offense	1	0	0	0	-	-
Traffic offense	2	0	0	7	4	57.1
Drunken stupor	0	-	-	10	2	20
Property offense	15	6	40	61	27	44.3
Resistance to state authority	1	0	0	1	1	100
Coercion, kidnapping	3	0	0	3	0	0
Threat	2	0	0	0	-	-
Robbery, extortion	5	0	0	24	4	16.7
Narcotics offense	0	-	-	201	44	21.9
Arson	19	1	5.3	10	2	20
Assault	37	5	13.5	75	19	25.3
Homicide, manslaughter	25	0	0	21	2	9.5
Total	110	12	10.9	415	105	25.3

Encouragingly, no capital offense in terms of homicide or manslaughter was committed in both patient samples after discharge. Moreover, the severity of the reoffenses decreased significantly compared to the severity of the index offenses in both samples (*Z* = - 1.971, *p* = .049 for patients placed according to Section 63; *Z* = 4.595, *p* <.001 for patients placed according to Section 64).

### Disorder-related recidivism rates

Within the group of patients placed according to Section 63, patients with a main diagnosis of mood disorder, mixed personality disorder, and conduct disorder comprised the highest general recidivism rates with at least half of the patients committing new offenses during the time at risk (see **Table 7**). They also more often exhibited relevant recidivism. However, incidence numbers for these disorders were comparatively low in this sample. Patients diagnosed with schizophrenia spectrum disorder and emotionally unstable personality disorder, who comprised the highest incidence numbers in this group (*n* = 79 and *n* = 12, respectively, see **Table 1**), generated general recidivism rates of about 17% each. However, recidivism rates increased to 53.8% in case of a schizophrenia spectrum disorder and a comorbid alcohol use disorder. Accordingly, the risk of general reoffending increased eightfold (Exp[*B*] = 8.167; *p* = .025), while a sole diagnosis with schizophrenia spectrum disorder reduced the risk of general recidivism (Exp[*B*] = .247; *p* = .024). Most violent reoffenses (*n* = 8) were committed by patients with a schizophrenia spectrum disorder, generating a violence recidivism rate of 10% (see **Table 7**). One patient with conduct disorder recidivated with a violent offense, resulting in a violent recidivism rate of 33.3% for this disorder.

**Table 7:** Disorder-related recidivism rates of patients placed according to Section 63.

Main diagnoses	*n*	General recidivism		Violent recidivism		Relevant recidivism	
		Frequency	%	Frequency	%	Frequency	%
Organic mental disorders (F0)	5	0	0	0	0	0	0
Schizophrenia spectrum disorders (F2)	79	14	17.2	8	10.1	6	7.6
*comorbid with alcohol use disorder (F10)*	*13*	*7*	*53.8*	*4*	*30.8*	*4*	*30.8*
Mood disorders (F3)	4	2	50	0	0	2	50
Neurotic and stress-related disorders (F4)	2	0	0	0	0	0	0
Personality disorders (F6)							
Dissocial (F60.2)	1	0	0	0	0	0	0
Emotionally unstable (F60.3)	12	2	16.7	0	0	2	16.7
Mixed (F61)	2	1	50	0	0	1	50
Intellectual disability (F70)	1	0	0	0	0	0	0
Conduct disorders (F91)	3	2	66.7	1	33.3	1	33.3
No diagnosis	1	0	0	0	0	0	0
Total	110	21	19.1	9	8.1	12	10.9

Most patients placed according to Section 64 were diagnosed with a substance use disorder as their main diagnosis (94.7%; *n* = 393; see **Table 2**). All other main diagnoses had a substance use disorder as a comorbid diagnosis.

The highest general recidivism rates were generated by patients with a main diagnosis of substance use disorder due to the use of sedatives and mixed personality disorder, who almost all recidivated, if only with low incidence numbers (see **Table 8**). Subsequently, patients with substance use disorders due to multiple drug use had the highest general recidivism rate of 49%. Upon closer examination, patients diagnosed with a substance use disorder and a comorbid personality disorder exhibited an even higher general recidivism rate (50.6%).

**Table 8:** Disorder-related recidivism rates of patients placed according to Section 64.

Main diagnoses	*n*	General recidivism		Violent recidivism		Relevant recidivism	
		Frequency	%	Frequency	%	Frequency	%
Substance use disorders due to…							
… use of alcohol (F10)	71	30	46.5	17	23.9	13	18.3
… use of opioids (F11)	73	33	45.2	6	8.2	22	30.1
… use of cannabinoids (F12)	10	3	30	1	10	1	10
… use of sedatives or hypnotics (F13)	3	3	100	0	0	2	66.7
… use of cocaine (F14)	4	1	25	0	0	1	25
… use of other stimulants (F15)	38	11	28.9	2	5.3	8	21.1
… multiple drug use (F19)	194	95	49.0	20	10.3	52	26.8
*Comorbid with a personality disorder (F6)*	*79*	*40*	*50.6*	*17*	*21.5*	*23*	*29.1*
Schizophrenia spectrum disorders (F2)	3	1	33.3	1	33.3	1	33.3
Mood disorders (F3)	1	0	0	0	0	0	0
Personality disorders (F6)							
Emotionally unstable (F60.3)	13	6	46.2	3	23.1	4	30.8
Mixed (F61)	5	4	80	1	20	1	20
Total	415	187	45.1	51	12.3	105	25.3

The highest violent recidivism rates were generated by patients with schizophrenia spectrum disorder (33.3%) and alcohol use disorder (23.9%), closely followed by patients diagnosed with a personality disorder (22.2%, see **Table 8**). Correspondingly, patients with a main diagnosis of substance use disorder and a comorbid personality disorder yielded a similar violent recidivism rate (21.5%). These patients also showed a fourfold increased risk of violent reoffending (Exp[*B*] = 4.204; *p* = .029), while patients who were solely diagnosed with a substance use disorder did not yield a statistically significant probability for violent recidivism (Exp[*B*] = .587; *p* = .096). Most relevant reoffenses, albeit with low incidence numbers, were committed by patients with a substance use disorder due to the use of sedatives and schizophrenia spectrum disorder.

### Subgroup analysis

About 65% of patients placed according to Section 64 completed treatment (*n* = 270), while one third of patients dropped out prematurely (*n* = 138). Seven patients were excluded from analysis due to imprecision concerning their completion of treatment.

Patients whose treatment ended because the maximum period of the placement order had expired (*n* = 55) were considered regularly discharged. These patients showed a general recidivism rate of 40% (*n* = 22), a violent recidivism rate of 10.9% (*n* = 6) and a relevant recidivism rate of 16.4% (*n* = 9).

Regularly discharged patients and treatment dropouts did not differ regarding the index offense or the distribution of disorders (see **Table 9**). However, patients who dropped out of treatment prematurely received significantly shorter sentences, had an earlier onset of criminal behavior as well as psychiatric symptoms, were more often in unstable relationships, had received more often drug substitution in the past and showed a more problematic course of treatment with higher amounts of relapses, violent outbursts, and other antisocial behavior during treatment. Accordingly, patients who were discharged prematurely showed a general recidivism rate of 65.2% (*n* = 90), while patients who were discharged regularly recidivated with 35.6% (*n* = 96; *χ²* (1) = 32.392, *p* <.001). Regarding violent recidivism, 7.8% (*n* = 20) of regularly discharged patients reoffended with a violent offense compared to 21.7% (*n* = 30) of prematurely discharged patients (*χ²* (1) = 16.275, *p* <.001). Similarly, treatment dropouts recidivated more often with relevant offenses (42%; (*n* = 58) than their counterparts (17.4%, *n* = 47; *χ²* (1) = 28.967, *p* <.001).

**Table 9:** Comparison of regularly and prematurely discharged patients placed according to Section 64.

	Regular discharge		Premature discharge		Statistics
	*n*	*n* (%)/ mean *(SD)*	*n*	*n* (%)/ mean *(SD)*	
Age at discharge (y)	270	36.80 (9.4)	138	33.18 (8.5)	*U*=14595.5***
Relationship status at discharge	268		138		*χ²* (3)=42.794***
Single		164 (61.2)		57 (41.3)	
Married		18 (6.7)		7 (5.1)	
Stable relationship/de-facto marriage		45 (16.8)		12 (8.7)	
Unstable relationship		41 (15.3)		62 (44.9)	
Main diagnosis at discharge	270		138		*χ²* (8)=11.091
Disorders due to use of alcohol		48 (17.8)		23 (16.7)	
Disorders due to use of opioids		43 (15.9)		28 (20.3)	
Disorders due to use of other substances		11 (4.1)		6 (4.3)	
Disorders due to use of other stimulants		29 (10.7)		8 (5.8)	
Disorders due to multiple drug use		124 (45.9)		66 (47.8)	
Schizophrenia spectrum disorders		3 (1.1)		0 (0)	
Mood disorders		1 (.4)		0 (0)	
Emotionally unstable personality disorder		10 (3.7)		3 (2.1)	
Mixed personality disorders		1 (.4)		4 (2.9)	
Age of onset of psychiatric symptoms (y)	270	14.71 (6.9)	138	13.49 (6.3)	*U*=16168.5*
Drug substitution	235	108 (46.0)	122	72 (59.0)	*χ²* (1)=5.479*
Relapses during treatment	270	67 (24.8)	138	65 (47.1)	*χ²* (1)=20.726***
Violence during treatment	270	21 (7.8)	138	18 (13.0)	*χ²* (3)=8.064*
Other antisocial behavior	270	127 (47.0)	138	96 (69.6)	*χ²* (1)=18.702***
Duration of treatment (y)	270	2.77 (.8)	138	1.31 (1.0)	*U*=5514.5***
Index offense	270		138		*χ²* (11)=18.147
Sexual coercion		1 (.4)		0 (0)	
Defamation, false allegation		1 (.4)		0 (0)	
Traffic offense		5 (1.9)		2 (1.4)	
Drunken stupor		7 (2.6)		3 (2.2)	
Property offense		32 (11.6)		28 (20.3)	
Resistance to state authority		0 (0)		1 (.7)	
Coercion, abduction		2 (.7)		1 (.7)	
Robbery, extortion		16 (5.9)		7 (5.1)	
Narcotics offense		145 (53.0)		53 (38.4)	
Arson		8 (3.0)		2 (1.4)	
Assault		40 (14.8)		34 (24.6)	
Homicide, manslaughter		13 (4.8)		7 (5.1)	
Violent index offense	270	86 (31.9)	138	55 (40.0)	*χ²* (1)=2.586
Index offense severity	270	3.29 (5.2)	138	3.61 (5.3)	*U*=17394.0
Sentence (months)	270	38.21 (22.0)	136	27.01 (17.1)	*U*=11853.0***
Age at first conviction (years)	270	26.64 (9.4)	137	23.27 (8.6)	*U*=14192.5***
Number of previous convictions	270	4.16 (3.8)	138	6.09 (4.9)	*U*=13978.0***
Time at risk (years)	270	6.20 (5.8)	138	3.24 (3.7)	*U*=10407.5***

*SD*, standard deviation; y, years; *U*, Mann-Whitney*-U; *χ*²*, Chi-square (Pearson); *p <.05, ***p <.001 (asymptotic, two-tailed).

While the severity of new crimes compared to the index offense dropped significantly for patients who completed treatment (*Z* = -4.469, *p* <.001), the severity of reoffenses for patients who dropped out of treatment did not (*Z* = -1.741, *p* = .082). They also showed a significantly shorter time at risk than regularly discharged patients (see **Table 9**).

**Figure 2:**
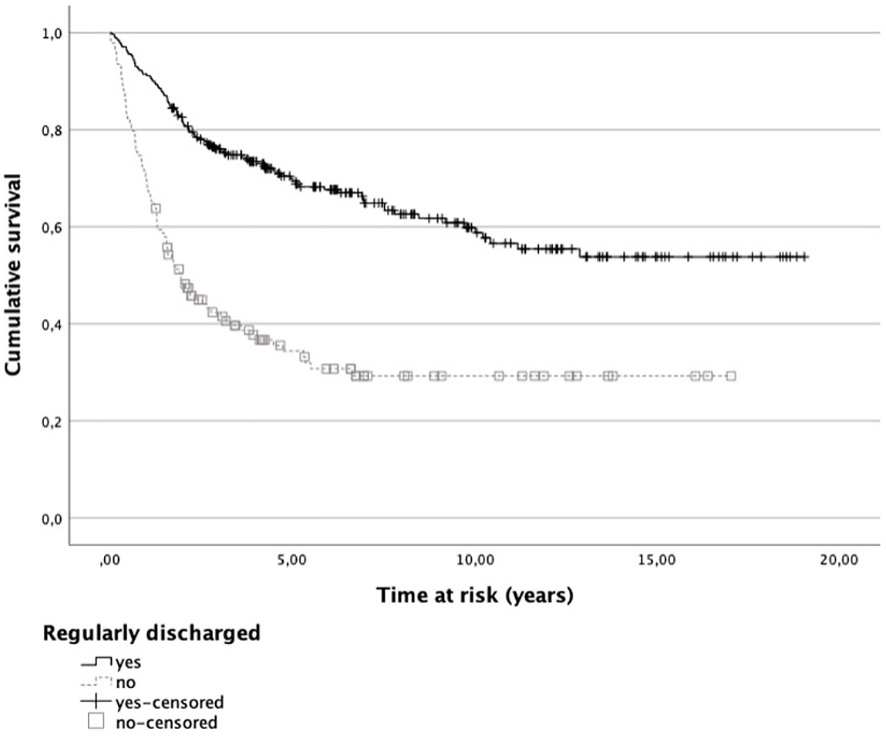
Survival functions for general recidivism of regularly and prematurely discharged patients. Note. Patients who did not recidivate within the time at risk are included as censored cases.

Accordingly, survival functions for general recidivism of regularly and prematurely discharged patients yielded a shorter time for non-recidivism for treatment dropouts, with 91.5% of regularly discharged patients not recidivating within the first year after discharge compared to 70.3% of treatment dropouts (see **Figure 2**). This difference grew over the course of the time at risk with 69.9% of regularly discharged patients not having recidivated within five years after discharge compared to 34.3% of prematurely discharged patients. Cox regression analysis showed that treatment dropouts were about 2.9 times more likely to recidivate during the time at risk than their counterparts (Exp[*B*] = 2.863; *p* <.001).

## Discussion

To our knowledge, this study is the first to report detailed recidivism data for female offenders with mental disorders in Germany. It provides not only general, violent, and relevant recidivism rates but also offense- and disorder related reoffending data following forensic psychiatric treatment, generating empirical data for appropriate risk assessment. With recidivism data being scarce and, if reported, mostly concerning male samples or samples with a vast majority of male subjects or an undisclosed gender distribution, risk assessment for female offenders usually relies on recidivism data for (largely) male samples (31). Recidivism rates presented in the current study can serve as a valuable empirical base for comprehensive gender considerations in risk assessment, contributing to the identification of gender-specific risk factors and informing the application of risk assessment instruments for women [e. g., the Female Additional Manual as a supplement to the HCR-20 Version 3; (8, 32)]. Moreover, in accordance with the Risk-Need-Responsivity framework (33), which in itself yielded a gender-specific risk assessment instrument [Women’s Risk Need Assessment; (34)], the results can also help develop gender-specific treatment strategies to reduce recidivism risk, answering a recent call for research in forensic psychiatry to improve treatment based on empirical findings (35).

With general recidivism rates of 19% and 45% for patients released from placement according to Sections 63 and 64, respectively, results of the study support the general finding that women recidivate less than their male counterparts (2, 12, 16, 17). They also substantiate the advocated need of gender-responsive risk assessment, as extant literature suggests that female offenders are often housed at higher security levels than necessary given their low recidivism rates, particularly for reoffending with violent and sex crimes (36, 37). Particularly, the subgroup analysis showed that treatment is associated with lower recidivism rates not only for male (17, 18) but also female offenders with substance use disorders.

The current study further showed that patients convicted of property offenses had among the highest general and relevant recidivism rates in both samples. Given that poverty has shown to be a risk factor for recidivism particularly among justice-involved women (38), it can be hypothesized that property offenses may stem from financial difficulties persisting after discharge. This finding may inform treatment plans on ensuring financial security after discharge.

For patients placed according to Section 63, albeit low incident numbers, patients who committed threats generated the highest recidivism rates regarding violent reoffending. This finding may correspond with existing literature showing that patients with schizophrenia spectrum disorder, who comprised the majority of this group, are more likely to escalate threats into violent actions compared to those with other mental disorders (39). Nevertheless, we did not find a significant correlation between a violent index offense and a violent reoffense in this group. This can be explained when considering that violence in patients with schizophrenia is usually preceded by psychotic symptoms like delusions (40) and that the reduction of these symptoms is not only a treatment goal but also a prerequisite for release (19). Yet, due to the low number of violent reoffenses in our study, violent recidivism rates should be interpreted cautiously for this group.

The findings for patients placed according to Section 64 are somewhat in line with recidivism rates of male patients (15) as well as female prisoners reported by Köhler (27), who found for women convicted of property, traffic and narcotics offenses to have the highest reoffending rates of 33-40%. In our sample, however, we also found patients who committed arson to have among the highest recidivism rate for general and the highest recidivism rate for violent reoffending. Correspondingly, female arsonists were found to struggle more often with substance use than both female offender controls (41) as well as male arsonists (42), suggesting that fire setting in females could represent a behavioral manifestation of a broader spectrum than in their male counterparts (43). High recidivism rates for various crimes, including violent offenses, indicate that treatment of this complex psychopathology is challenging (44).

We generally found a significant correlation between a violent index offense and a violent reoffense for patients placed according to Section 64. This finding may be attributed to coinciding risk factors for violence exhibited by women with substance use disorders like traumatic experiences, dysfunctional relationships, and personality disorders, resulting in refractory violent behavior patterns (45, 46).

As detailed reports on disorder-related recidivism rates are especially scarce, comparison with previous research is limited. Compared to male samples, our study overall showed lower recidivism rates especially for patients diagnosed with schizophrenia as well as substance use disorders (12–14). For further analyses, we focused on the comorbidities that were both most prevalent in our study as well as highly scrutinized in extant recidivism and risk assessment literature (13), i. e., alcohol use disorder and personality disorders. While patients placed according to Section 64 were about three times more likely to recidivate, we found for both groups that about every third patient diagnosed with a comorbid schizophrenia spectrum disorder and a substance use disorder, more specifically alcohol use disorder in the case of patients placed according to Section 63, recidivated with a violent offense. A single diagnosis of schizophrenia spectrum disorder, however, decreased the probability of reoffending. Suitably, in a recent meta-analysis on recidivism of justice-involved women with mental disorders, psychosis was not supported as a predictor of general recidivism (47).

Among patients placed according to Section 64, one in five with a comorbid substance use disorder and a personality disorder committed a violent reoffense, representing a fourfold increase in the risk of violent recidivism compared to those diagnosed solely with a substance use disorder. Our results suggest that, if treated, both sole schizophrenia spectrum disorder and substance use disorder diagnoses do not impose a significant recidivism risk. This substantiates existing literature on the cruciality of comorbidities when it comes to recidivism of both justice-involved men (13) and women with mental disorders (48). Thus, the results help differentiate the impact of overarching categories of psychopathology that trigger greater concern for recidivism than other types of mental disorders, cutting against the public narrative that severe mental disorders like schizophrenia imperatively yield crime and recidivism (49).

The results of the present study overall indicate that treatment reduces not only the rate but also the severity of recidivism in females with mental disorders. Not only was no capital offense committed in both patient samples after discharge, but the severity of the reoffenses significantly decreased compared to the index offenses in both samples. This is also mirrored by the finding that most recidivists were sentenced to a money fine for the first reoffense.

While previous research as well as the current study suggest treatment to be overall effective, forensic psychiatric care also draws on scarce financial and personal resources, which often are even more strained due to overcrowding in forensic psychiatric hospitals, leading to a precarious treatment situation in Germany (50). Yielding patient characteristics of treatment dropouts, the results of this study can contribute to a more effective and concrete clinical assessment and judicial decision making on the likelihood of a favorable treatment prognosis. In particular, we found that treatment dropouts were subject to comparatively shorter sentencing, exhibited an earlier onset of criminal behavior as well as psychiatric symptoms, including a higher incidence of prior drug substitution, demonstrated a more challenging treatment trajectory characterized by increased rates of relapses, violent episodes, and other antisocial behaviors, and were more frequently involved in unstable relationships. The results suggest that these patients not only exhibit more severe psychiatric symptoms but also underwent extended and intensified socialization within a dissocial environment, as indicated by the higher incidence of prior participation in drug substitution programs (51). Given the shorter sentences, the available treatment time of approximately two years appears to be too short to adequately address the symptomatology and rehabilitation needs of these both “mad and bad” (52) patients. This might be reflected by the slightly higher general and violent recidivism rates of patients who were discharged due to the expiration of the maximum period of the placement order (40% and 10.9%, respectively) compared to the entire group of regularly discharged patients (35.6% and 7.8%, respectively), suggesting that some of these patients might have benefitted from a longer treatment duration. Nevertheless, the group of regularly discharged patients seems to be “healthier”, with better adjustment skills and fewer risk factors, rendering them more responsive to treatment and consequently yielding more positive and prognostically favorable outcomes within the given timeframe for treatment. Moreover, since treatment dropouts showed a more profound criminal history with an earlier onset of criminal behavior and a higher number of previous convictions, they might have exhibited a potentially higher recidivism rate at baseline (53). This probable selection bias needs to be considered when interpreting our results (9). Nevertheless, our study showed lower dropout-rates than research with male samples (15–17), substantiating previous findings (9) as well as the well-established understanding of women being more receptive to psychiatric treatment than men (54).

Survival analyses of recidivism following discharge showed that patients placed according to Section 63 exhibited an increase in reoffenses after approximately five years post discharge. For patients placed according to Section 64, on the other hand, most reoffending occurred during the first five years post discharge. As conditional release regularly involves mandatory supervision and forensic aftercare for five years (Section 68 of the German Criminal Code), these results underline the importance of forensic aftercare with respect to the prevention of recidivism. For patients placed according to Section 63, with the majority being diagnosed with schizophrenia spectrum disorder, effective rehabilitation appears to demand a prolonged treatment approach extending beyond the legally mandated forensic aftercare for this group. The inherent deficits in the psychosocial functioning of schizophrenia patients, exacerbated by progressive chronification and potential discontinuation of medication intake or abstinence from substances, underscore the need for additional and continuous aftercare (14). Additionally, more recent research indicates that the support provided by structured and intensive outpatient settings and forensic aftercare contributes to reducing the risk of reoffending among female patients not only with schizophrenia, but also substance use disorders (9). In the case of patients placed according to Section 64, this could also have contributed to our finding of an almost three times higher recidivism risk for treatment dropouts compared to regularly discharged patients who were ordered to mandatory forensic aftercare, as the latter profit from support in their social rehabilitation, including recidivism risk reducing factors like the reintegration into the workforce and stable living conditions post-discharge (14, 55), both during treatment and through forensic aftercare. Nevertheless, while the bulk of knowledge regarding recidivism originates from studies on male offenders, which may not fully or accurately explain the post-discharge trajectories of female offenders resulting in recidivism (56), more prospective research on post-discharge experiences of female patients is needed in order to account for changing living conditions as well as mental health trajectories and their association with recidivism.

### Future directions

Considering the recent statutory changes to both Sections 63 and 64 of the German Criminal Code to counteract the precarious conditions in German forensic psychiatric hospitals within the legal framework (50, 57), prospective studies should also analyze the impact of these changes on recidivism rates of female offenders. Specifically, since the amendment to Section 63 of the German Criminal Code, enacted on August 1, 2016, aims at reducing lengths of stay through stricter adherence to proportionality of placement duration compared to the severity of the expected reoffenses, it is also presumed to impact recidivism rates when patients are being discharged against clinical recommendations (14). While no patients were released due to proportionality ruling in our sample, first reports indicate an unfavorable legal prognosis for this group in (largely) male samples (14, 58). Moreover, on October 1, 2023, the amendment to Section 64 of the German Criminal Code was implemented to significantly reduce placement numbers through more precise statutory requirements regarding the presence and severity of a substance use disorder, its relevance to the committed offense, and the likelihood of a favorable treatment outcome (59). Initial research indicates that both patient as well as treatment dropout numbers can be reduced when strictly adhering to the amended placement requirements (57). Whether the amended requirements succeed in mitigating overcrowding, “misplacement” of patients (15), and reoffending, in particular of female patients, has yet to be examined. The results of the current study, however, can help counteract above mentioned problems and support the implementation of the amendment by presenting patient characteristics associated with premature discharge, which in turn can enable clinical professionals to identify potential treatment dropouts and inform judicial authorities on the likelihood of a favorable treatment prognosis.

### Limitations

Following limitations should be considered when interpreting the results of this study and drawing conclusions for their use in risk assessment. First, as a retrospective study, data collection was based on preexisting file information after patients had already been discharged. This means that missing data could not be reexamined, and the accuracy of the information could not be verified. Also, low incidents numbers in some of the offense- and disorder-related data as well as the lack of a control group restrict their interpretability. However, the lack of adequate control groups is a common shortcoming in research on forensic psychiatric populations (60). We also tried to partially overcome this shortcoming by analyzing the efficacy of the treatment with treatment dropouts serving as an approximate control group.

Further, we measured recidivism based on official conviction records provided by the German Federal Office of Justice. As there are various reasons for why an official record entry may be delayed or not made at all, the actual recidivism rates may have been higher (61). Future study designs could therefore additionally incorporate self-reported and third-party obtained reoffending information, for example through probation officers or aftercare staff, as well as international records and records of arrests.

Moreover, limiting data collection to a single forensic hospital restricts generalizability and sample size. Additionally, the variability of treatment and discharge practice between different federal states coupled with disparities between legal prerequisites for forensic psychiatric treatment in Germany and other countries reduces comparability with both national and international studies. However, focusing on a single hospital also bears methodological benefits such as consistency of record keeping procedures and substantial reduction of external influencing factors due to differing regional and national treatment conditions.

### Conclusion

Notwithstanding its limitations, the present study makes a significant contribution to the limited body of literature concerning recidivism rates for female offenders with mental disorders. Primarily, it addresses methodological deficiencies of previous research, such as small sample sizes and short follow-up periods. Notably, the included sample represents a comprehensive survey of all female patients within a forensic psychiatric hospital in Germany, spanning a total of 17 years. To our knowledge, this study produced two of the largest sample sizes in recidivism research on forensic psychiatric patients in Germany. Our findings therefore not only hold particular relevance within a forensic context, but also provide rare empirical data for informing risk assessment and treatment strategies for the understudied group of female offenders with mental disorders.

## Data Availability

The raw data supporting the conclusions of this article will be made available by the authors, without undue reservation.
